# Anthropogenic noise pollution from pile-driving disrupts the structure and dynamics of fish shoals

**DOI:** 10.1098/rspb.2017.1627

**Published:** 2017-09-27

**Authors:** James E. Herbert-Read, Louise Kremer, Rick Bruintjes, Andrew N. Radford, Christos C. Ioannou

**Affiliations:** 1 Department of Zoology, Stockholm University, 10691, Stockholm, Sweden; 2 Department of Agronomy, Agroequipments, Farming and Environment, AgroSup Dijon, Dijon, France; 3 Biosciences, College of Life and Environmental Sciences, University of Exeter, Exeter, UK; 4 School of Biological Sciences, University of Bristol, Bristol, UK

**Keywords:** noise, collective behaviour, global change, shoaling, pile-driving

## Abstract

Noise produced from a variety of human activities can affect the physiology and behaviour of individual animals, but whether noise disrupts the social behaviour of animals is largely unknown. Animal groups such as flocks of birds or shoals of fish use simple interaction rules to coordinate their movements with near neighbours. In turn, this coordination allows individuals to gain the benefits of group living such as reduced predation risk and social information exchange. Noise could change how individuals interact in groups if noise is perceived as a threat, or if it masked, distracted or stressed individuals, and this could have impacts on the benefits of grouping. Here, we recorded trajectories of individual juvenile seabass (*Dicentrarchus labrax*) in groups under controlled laboratory conditions. Groups were exposed to playbacks of either ambient background sound recorded in their natural habitat, or playbacks of pile-driving, commonly used in marine construction. The pile-driving playback affected the structure and dynamics of the fish shoals significantly more than the ambient-sound playback. Compared to the ambient-sound playback, groups experiencing the pile-driving playback became less cohesive, less directionally ordered, and were less correlated in speed and directional changes. In effect, the additional-noise treatment disrupted the abilities of individuals to coordinate their movements with one another. Our work highlights the potential for noise pollution from pile-driving to disrupt the collective dynamics of fish shoals, which could have implications for the functional benefits of a group's collective behaviour.

## 1. Introduction

Human activities, such as urbanization, resource extraction, transportation, and energy production, generate considerable noise. Since the Industrial Revolution, these human-generated noise sources have resulted in major changes in soundscapes across the globe, both due to an increase in sound levels and the addition of sounds that are different from those arising from natural sources [1–3]. Consequently, anthropogenic noise is now recognized as a pollutant of international concern, being included in legislation such as the US National Environment Policy Act and the European Commission Marine Strategy Framework Directive. To inform policymakers, to develop effective management strategies, and to design suitable mitigation methods, detailed information on the organismal impacts of anthropogenic noise are needed.

There is mounting experimental evidence that anthropogenic noise can have a variety of negative physiological and behavioural effects on individual animals, ultimately affecting their survival and reproductive success [4–7]. For example, noise from human activities can directly cause injury or hearing loss in some species [8,9], or as a consequence of masking (i.e. reducing the signal-to-noise ratio), can impair the ability of animals to communicate [10–12]. Further, noise can be perceived as a threat, be a distraction, or can cause increased stress, in turn impairing an animal's ability to forage efficiently [13,14], respond appropriately to information about predation risk [15–17], perform adaptive behaviours during habitat-selection [18,19], or reproduce successfully [20,21]. However, despite the abundance of group-living species, there has been relatively little research on how the social behaviour of animals is affected by anthropogenic noise (but see [22–26]). An understanding of noise effects in this regard is crucial because many animals rely on such behaviours for their survival and reproductive success [27,28], and often adjust their social behaviour in response to risk [29,30]. Thus, any potential impacts of noise on social behaviour could have fundamental ecological and evolutionary implications for social species.

The impacts of anthropogenic noise can be particularly prevalent in aquatic environments, where sound travels further and faster before attenuation than in air [31]. Fish, in particular, are known to be affected by noise in a variety of ways [6,32]. For example, acoustic communication between individuals may be disrupted in the presence of noise, fish may move away from noisy sound sources, and in extreme circumstances, noise can even result in injury or death [6,32]. There has also been recent experimental evidence that anthropogenic noise can negatively affect the foraging [14,33], anti-predator [16,34], and parental care [21,23] behaviour of individual fish. It is estimated that approximately 50% of fish species form shoals during their lifetimes [35], with juvenile fish regularly shoaling in inshore areas [36] which are often subject to noisy exploration and construction projects [37]. Shoaling is achieved when individuals use simple interaction rules, including speed and direction changes, to coordinate their movements with near neighbours [38–40]. Information about neighbours' movements and positions is acquired through the lateral-line and visual systems [41,42], and there are good reasons to suspect that noise generated by human activity might affect shoaling dynamics.

Noise could impact the ability of individuals to coordinate their movements by masking information about neighbours' positions that could have been detected through the lateral line (uni-modal effects). Alternatively, or in addition to masking effects, distraction or stress could impair the coordination of individuals' movements by compromising an individual's ability to process information in another sensory channel (i.e. vision or olfaction), otherwise known as ‘cross-modal’ effects [43]. There is some evidence that noise produced by motorboats can affect the shape and structure of bluefin tuna (*Thunnus thynnus*) schools [22], and this could impair two of the key benefits of shoaling. First, shoaling provides anti-predatory benefits through dilution, confusion, and selfish-herd effects, with individuals in larger, more cohesive groups having proportionally less risk than individuals in smaller, less cohesive groups [44–47]. Changes in the cohesion of groups, therefore, could act to increase predation risk. Second, shoaling provides individuals access to social information, whereby information about detected threats or resources can be gathered by copying the movement decisions of others [48–51]. Disruption to the abilities of individuals to copy these decisions could have considerable implications for how individuals in groups detect resources or avoid predators. High-resolution data on the positions and movements of individuals in shoals are needed, therefore, to measure how individuals are interacting in groups, and hence how anthropogenic noise may affect these interactions.

Here, we use a laboratory-based experiment to ask how anthropogenic noise (specifically playback of pile-driving noise, an impulsive sound source) impacts the shape, organization, and dynamics of European seabass (*Dicentrarchus labrax*) shoals. Seabass are known to be affected by playbacks of anthropogenic noise [52,53], making them a model species to use in these experiments. Laboratory-based experiments cannot perfectly replicate real-world sound fields or natural behaviour [54,55], but they allow tight control of other variables [55], as well as the collection of detailed (high spatial and temporal resolution) tracking data on shoaling behaviour, which has only recently been recorded in the wild [56]. We predicted that if the additional noise was perceived as a threat [57], the seabass would form denser, more directionally ordered shoals, with increased coordination of speed and direction changes, in the pile-driving treatment compared to an ambient-sound control treatment. If, however, the additional noise masks important information, or causes stress or distraction [4,58], the seabass would be predicted to form less cohesive and directionally ordered shoals, with reduced directional and speed coordination, in the pile-driving treatment compared to times with ambient-sound playback.

## 2. Material and methods

### (a) Experimental subjects

Juvenile sea bass were sourced from Ifremer (Plouzane, France) and transported to the University of Exeter, where they were held for two months before being transported to the University of Bristol aquarium facilities. The fish were held in 40 × 70 × 34 cm and 20 × 70 × 34 cm (width × length × height) 5 mm glass stock tanks that contained artificial plants. Fish were generally fed daily on a uniform commercial fish food diet (Perla MP Pellet, Skretting, Norway) except during a 7-week period in February–March 2015 when half of them were only fed three times per week. In this study, fish were randomly allocated to the sound treatments regardless of this feeding regime difference. Water temperature was 15.7 ± 0.2°C; lighting was kept on a 12 L : 12 D cycle; salinity was maintained between 35 and 36 parts per thousand (ppt). Experiments were conducted in July 2015 when the fish measured 9.7 ± 0.7 cm (mean ± s.d.) standard body length. The size of the fish did not differ between treatments (see below; Linear Model (LM): *F*_1,118_ = 0.10, *p* = 0.75).

### (b) Recordings and playbacks

Original field recordings of offshore pile-driving in Swansea Bay, UK, were made between 87 and 200 m from the sound source [52,53]. Pile-driving at this site involved a 1.2 m diameter monopole being driven around 25 m into the seabed at a water depth of 6.5 m. The recordings of this process were made with a Hi Tech Inc. HTI-99HF hydrophone with inbuilt preamplifier (manufacturer calibrated sensitivity −204 dB re 1 V μPa^−1^, 20–125 000 Hz frequency range) and a data logger (RTsys EASDA, 44.1 kHz sampling rate). Recordings of ambient coastal sound were made at Portsmouth, Plymouth, and Gravesend, UK, using a Hi Tech Inc. HTI 96-MIN hydrophone with inbuilt preamplifier (manufacturer calibrated sensitivity −164 dB re 1 V μPa^−1^, 20–30 000 Hz frequency range) and a digital recorder (Roland Edirol R09HR 24 bit, 44.1 kHz sampling rate) [52,53]. For ambient-sound recordings, the hydrophone was positioned at 1 m depth 20–40 m offshore. All recordings were made during low to moderate wind speeds.

The original recordings of pile-driving noise and ambient sound were used to create three tracks per sound treatment; a random part of the relevant recording was used in each case (as in [52,53]). Multiple playback tracks per sound treatment were used to reduce pseudo-replication issues. All ambient playback tracks were 5 min in duration, and the pile-driving playbacks were 10–30 s in duration, with each track looped for the 5 min playback period. All the pile-driving playbacks had a pile-driving rate (time between pile-driving events) of 1.7 s. All tracks were created using Audacity 1.3.13 (http://audacity.sourceforge.net/).

Tracks were played back via an underwater loudspeaker (Aqua30; frequency range 80–20 000 Hz: www.dnh.no), an amplifier (Kemo Electronics GmbH; 18W; frequency response range approx. 40–20 000 Hz), and a laptop (Toshiba Portege R930-1CW), as in [34,52,53]. To measure the recordings of the playbacks and any ambient sound in the room, we placed a hydrophone (HTI 96-MIN) in the middle of the experimental tank, 5 cm above the tank bottom. Recordings of the sound during the trials were made using a digital sound recorder (Sony PMC-M10, 44.1 kHz sampling rate) connected to the hydrophone. Because of unresolved challenges in measuring particle motion in small tanks at the time of the experiment, acoustic conditions were assessed in the sound-pressure domain only. In this experiment, we do not establish absolute values for sensitivity, but rather explore the potential impact of the change in additional sound on the fish's behaviour (see also, for example, [34,52,53]).

### (c) Acoustic analysis

All sound recordings were analysed in MATLAB (v. 2013a and 2017a) using pamGuide [59] and paPAM [60]. Spectrograms (see the electronic supplementary material, figure S1) were calculated for 1–2 000 Hz (the frequencies most likely to be of relevance to seabass [61]) using a Hann evaluation window, 50% overlap, 0.1 s window length over 20 s recordings. Cumulative sound exposure level (SELcum) was calculated for the whole 5 min exposure period, whereas sound-pressure level (SPL) was calculated over 20 s recordings (electronic supplementary material, table S1). Zero-to-peak level, 90% energy envelope, rise time, and single-strike sound-exposure level (SELss) were calculated using an average of five randomly selected pile strikes (electronic supplementary material, table S1).

### (d) Experimental protocol

Trials took place in an octagonal arena located at one end (10 cm from the wall) of a 2.5 × 1.25 m aluminium tank lined with a white plastic PVC pond liner (electronic supplementary material, figure S2). The arena was made of white 68 × 43.4 cm Perspex panels, so the narrowest width of the arena was 105 cm. The loudspeaker was located at the other end of the tank, 20 cm from the wall facing the arena and half way along the width of the tank. The loudspeaker was suspended using string to be 2 cm above the bottom of the tank. Water depth was 10 cm and temperature and salinity conditions matched those in the stock tanks. The whole tank was covered by a cuboid frame and white sheeting to minimize disturbance and diffuse overhead fluorescent lighting to minimize reflections on the water surface. A Panasonic X920 camcorder, positioned centrally and 193 cm above the water's surface, was used to film the arena at a resolution of 1 920 × 1 080 pixels and frame rate of 59 frames per second.

Our focus in this study was the effect of exposure to additional noise; comparisons were made to individuals that experienced control playbacks (of recordings of ambient coastal noise) but were otherwise from the same cohort and held under the same conditions. Four fish were netted from the same stock tank and transferred gently to the test arena. Juvenile seabass generally occur in small group sizes [62], and we chose groups of four fish as this is within the range of group sizes used in previous studies on these fish [63–65]. The groups of four fish were given 15 min to acclimatize, during which time no playback occurred, with the last 5 min of this period filmed (termed ‘1st half of trial’ hereafter). One of the two sound treatments (pile-driving noise; *n* = 15 groups, or ambient sound; *n* = 15 groups) was then played to the fish for 5 min (termed ‘2nd half of trial’ hereafter), with filming continuing during this period. The trial order of sound treatments was determined by a complete random block; i.e. for each pair of trials (1st and 2nd, 3rd, and 4th, etc.), one of each treatment was given, but in a random order within that pair. Which of the three replicate recordings was used for each treatment was randomly determined. Each fish was tested only once. Fish were not fed on the day of testing until after the trials.

### (e) Response measures

The 10 min videos from each trial were converted to MPEG-4 with Handbrake 0.10.5 (https://handbrake.fr/). idTracker [66] was used to track the *x* and *y* coordinates of each fish throughout each trial. All subsequent analyses were performed using MATLAB (2016a) and followed similar methods to [67–69]. The parameters associated with the spatial and directional organization, as well as the movement dynamics of the fish, are detailed below. Measures were calculated separately for each fish in the group. All variables were calculated for the 1st (no playback) and 2nd (playback) half of each trial separately.

#### (i) Spatial and directional organization of the shoals

We first calculated measures associated with the cohesiveness of shoals including the mean distance each individual was to the shoal's centroid, and the modal nearest-neighbour distance of each individual. The modal nearest-neighbour distance represents the distances that pairs of individuals are most commonly observed apart [39,68]. We then calculated the distance from each fish to its nearest-neighbour perpendicular to their direction of travel (i.e. how far apart side-by-side) and parallel to the direction of travel (i.e. how far apart in front-or-behind one another). We further calculated the bearing angle to a fish's nearest neighbour, which represents the direction that a neighbour was most likely to be found in relation to the focal individual [38]. We treated bearing angles to the neighbour ahead or behind of the focal fish separately for ease of interpretation in the statistical models. A bearing angle of 90° would represent a neighbour that was directly to the side of a focal individual, whereas a bearing angle of 0° or 180° would represent a neighbour that was, respectively, directly in front or behind a focal individual. We also calculated the heading difference between nearest neighbours, i.e. the angle between the direction nearest neighbours were facing. This measures how closely aligned nearest neighbours in the shoals were, effectively measuring their directional organization [70]. The heading difference ranges from 0° (individuals were facing in the same direction = high alignment) to 180° (individuals were facing in opposite directions = low alignment). Full details of these calculations can be found in the electronic supplementary materials.

#### (ii) Movement dynamics of individuals in the shoals

The above measures determine the spatial organization of fish shoals, but do not capture how individuals are moving and interacting within them. To assess how individuals were moving and interacting in the shoals, we first calculated the speed and direction of each fish at each time point. From these values, we then calculated the cross-correlations between an individual's speed (or direction) and its nearest-neighbour's speed (or direction). Cross-correlations assess how strongly pairs of individuals copy each other's speed or direction changes in time, and we followed established methods described by Nagy *et al.* [71]. In brief, we identified the peak of the correlation in speed (or direction) and the time delay between when two individuals' speeds (or directions) were most strongly correlated. Higher peaks of the cross-correlation indicate the two fish were more strongly correlated in speed (or direction), and shorter absolute time delays indicate that the two individuals direction were more synchronized, with individuals adopting the speed or direction changes of their partner sooner. Because individuals' speeds (but not direction) were highly correlated in time (i.e. showed minimal time delays to the peak correlation) we did not analyse the time-delays between nearest-neighbours' speeds, but did analyse these delays for direction changes. Full details of these calculations can be found in the electronic supplementary materials.

### (f) Statistical analysis

All measures calculated from the fish trajectories were analysed as response variables in Mixed Models. All models included the treatment (pile-driving or ambient-sound playback) as a between-subjects term, and the half of the trial (1st or 2nd) as a within-subjects term. The interaction between these two fixed terms was included in the initial models, but was removed where it was non-significant and models were re-run with main effects only. All models included fish identity nested within trial (which is equivalent to the group the individuals belonged to) as the random term. The average difference between fish perpendicular or parallel to their direction of travel, the bearing of the nearest neighbour in-front or behind, and the difference in heading between nearest neighbours were analysed for each fish using negative binomial Generalized Linear Mixed Models (GLMMs), as the data were typically right skewed. For all GLMMs, the dispersion parameter was checked to be approximately equal to 1 (more than 0.5 and less than 2) using Generalized Linear Models with the same model structure but without the random effects. The variance function for the negative binomial models is *μ* × (1 + *μ*/*k*), for *k* > 0 (i.e. variance is approx. equal to the mean for *μ*≪*k* and proportional to the mean squared for *μ*≫*k*), where *μ* is the mean and *k* is the shape parameter for a negative binomial distribution.

The remaining response variables were analysed using Linear Mixed Models (LMMs). The median speed and the mean time delay that maximized the directional correlation with the nearest neighbour of each fish were analysed without transforming these response variables. The mean distance to the centroid and modal nearest-neighbour distance for each fish were log_10_ transformed before analysis. The mean maximum speed correlation and mean maximum directional correlation with the nearest neighbour for each fish were transformed by subtracting the correlation from one and then applying a log_10_ transformation (i.e. log_10_(1−correlation coefficient)). For all LMMs, the residuals from each model were checked to ensure normality (using QQ plots) and homoscedasticity (using the residuals plotted against the fitted values). The variance function here is Var(*μ*) = 1. Owing to correlations between speed and maximum speed and directional correlations, and between speed and the time delay that maximized the directional correlation, the models that analysed these response variables were repeated with the fish's speed as an additional main effect to control for the correlation of speed with the response variables.

## 3. Results

### (a) Spatial and directional organization of the shoals

**Figure 1.**
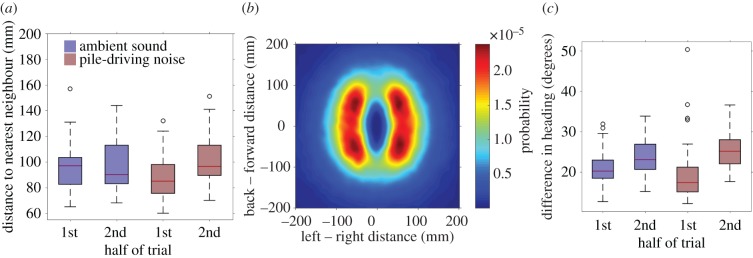
(*a*) Modal nearest-neighbour distances of fish during the 1st (no playback) and 2nd (playback) half of the trials. (*b*) The relative positions of a fish's nearest neighbour, combined for all fish and across both sound treatments. The focal fish is located at the origin of the plot and is facing along the positive *y*-axis. The heat in the plot shows the probability of finding the focal fish's nearest neighbour in locations surrounding the focal fish. ‘Hotter’ regions indicate a larger probability of finding a neighbour in that location. For plotting purposes, the heatmap has been smoothed with a Gaussian filter, *σ* = 6. (*c*) Mean angular difference in heading between a fish and their nearest neighbour in the 1st and 2nd half of the trial. In (*a*,*c*), blue bars represent the ambient-sound treatment and red bars represent the pile-driving treatment. Edges of each of the boxes represent the 25 and 75% percentiles, whiskers extend to all included data, black circles represent outliers.

The spatial structure of the shoals changed in both the ambient-sound and pile-driving playbacks. There was an interaction between treatment and the half of the trial when investigating group cohesiveness (LMM: *F*_1,118_ = 4.44, *p* = 0.04; electronic supplementary material, table S2a). When the ambient-sound playback was initiated, the mean distance of individuals to the group's centroid decreased, whereas this distance increased when the pile-driving playback treatment was initiated. Similarly, the modal nearest-neighbour distance decreased in the ambient-sound playbacks, whereas this distance increased in the pile-driving playbacks (interaction between treatment and trial half: *F*_1,118_ = 14.88, *p* < 0.001; figure 1*a*; electronic supplementary material, table S2b).

To investigate in more detail how the distances between fish in the pile-driving playback increased, we assessed the relative positions that individuals adopted next to their nearest neighbour. Individuals tended to position themselves in a lattice formation, with nearest neighbours most frequently being found at either 43° or 133° in front or behind the focal fish, respectively, and to the left or right, rather than directly in front or behind one another (figure 1*b*). During both playback treatments, the bearing angle to the nearest neighbour moved closer to 90°, indicating that fish were more likely to be observed side-by-side compared to before the playbacks were initiated (GLMM, effect of trial half on angle to neighbour in-front: *χ*^2^ = 11.22, d.f. = 1, *p* < 0.001; electronic supplementary material, table S2c; and angles to neighbour behind: *χ*^2^ = 12.78, d.f. = 1, *p* < 0.001; electronic supplementary material, table S2d). The distance between fish in this direction (i.e. the distance fish were apart perpendicular to their direction of travel) increased in both the ambient-sound and pile-driving playbacks, but this effect was larger in the pile-driving playback than the ambient-sound playback (interaction between treatment and trial half: *χ*^2^ = 7.72, d.f. = 1, *p* < 0.01; electronic supplementary material, table S2e).

The angular difference in heading between nearest neighbours also increased in both treatments, but this effect was larger in the pile-driving playback than in the ambient-sound playback (GLMM, interaction between treatment and trial half: *χ*^2^ = 7.99, d.f. = 1, *p* < 0.01; figure 1*c*; electronic supplementary material, table S2g).

### (b) Movement dynamics of individuals in the shoals

**Figure 2.**
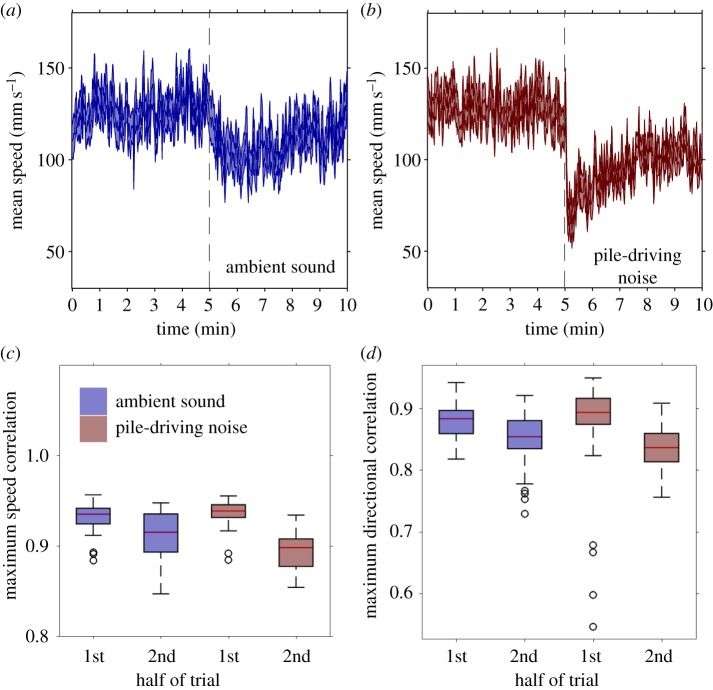
Mean speed (± s.e.) of fish in (*a*) ambient sound or (*b*) pile-driving noise treatment during the 1st half of the trial (no playback) and the 2nd half of the trial (playback). The dashed lines at 5 min separates the ‘no playback’ and ‘playback’ regions. (*c*) Mean maximum cross-correlation in speed between nearest neighbours in the 1st and 2nd half of the trials. (*d*) Maximum directional correlation between a fish and its nearest neighbour in the 1st and 2nd half of the trial. In each plot, blue bars represent the ambient-sound treatment, whereas red bars represent the pile-driving treatment. Edges of the boxes represent the 25 and 75% percentiles, whiskers extend to all included data, black circles represent outliers.

The speed of fish decreased when both the ambient-sound and pile-driving playbacks were initiated, but this effect was larger in the pile-driving playback (LMM, interaction between treatment and half of trial: *F*_1,118_ = 32.53, *p* < 0.001; figure 2*a*,*b*; electronic supplementary material, table S3a). The maximum correlation between nearest-neighbours' speeds also decreased in both playback types, but again this effect was larger in the pile-driving playback (interaction between treatment and half of trial: *F*_1,118_ = 46.43, *p* < 0.001; figure 2*c*; electronic supplementary material, table S3b). Repeating the statistical model with speed included as a covariate did not change this finding (*F*_1,132_ = 20.61, *p* < 0.001; electronic supplementary material, table S3c).

The maximum correlation between nearest-neighbours' directions in time decreased when the playbacks were initiated, but this effect was stronger in the pile-driving playback (LMM, interaction between treatment and trial half: *F*_1,118_ = 11.14, *p* < 0.01; figure 2*d*; electronic supplementary material, table S3d). This result held when including speed as a covariate in the model (*F*_1,132_ = 4.23, *p* = 0.04; electronic supplementary material, table S3e), indicating that larger reductions in speed in the pile-driving treatment could not solely explain this result. The time delay between nearest-neighbours' maximum directional correlations also increased more in the pile-driving playback than in the ambient-sound playback (interaction between treatment and trial half: *F*_1,118_ = 14.57, *p* < 0.001; electronic supplementary material, table S3f). However, this result could be explained based on the larger reductions in the speed of fish during the pile-driving playback (interaction between treatment and trial half when speed included as a covariate: *F*_1,132_ = 2.65, *p* = 0.11; electronic supplementary material, table S3g). In other words, larger reductions in speed in the pile-driving playback also caused larger delay times between nearest-neighbours' maximum directional correlations.

## 4. Discussion

Both pile-driving and ambient-sound playbacks affected the spatial and directional organization, as well as the coordination of seabass shoals, but these effects were often more pronounced when there was additional anthropogenic noise. In particular, the distance between fish increased more, and the directional and speed organization of the shoals decreased more during the pile-driving playbacks compared with the ambient-sound playbacks. Noise from the pile-driving treatment therefore caused significant changes to how individuals coordinated their movements with near neighbours, ultimately affecting the structure of the shoals. While most studies have concentrated on individual behavioural responses to anthropogenic noise, this study provides conclusive evidence that the social interactions of individuals within groups are also impacted by added noise.

Our experiment using both ambient-sound and pile-driving playbacks highlights that both sound types impacted the structure and dynamics of fish schools. The simple addition of sound beyond current baseline levels, therefore, impacts the shoaling behaviour of fish regardless of its source (i.e. ambient sound or pile-driving noise). Indeed, across sensory modalities, sensory systems are highly responsive to sudden changes in background conditions [72], as this reflects information about changes in the environment. Changes in behaviour in both treatments, such as reductions in speed, may therefore reflect increased alertness due to changes in environmental conditions. This highlights the importance of relevant controls that should be used during these types of playback experiments. Changes in the fish's behaviour between the 1st half of the trial (no playback) to the 2nd half of the sound (playback), however, were typically much larger in the pile-driving compared to the ambient-sound playback. When the ambient-sound and pile-driving playbacks changed the behaviour of the fish in the same direction, all effect sizes of these changes (comparing changes in the behaviour of the fish between the 1st and 2nd half of the trials) except one ranged between 0.75 and 1.18 (see electronic supplementary material, table S4), indicating the differences between the playbacks were medium to strong effects [73]. Neo *et al.* [65] found that fish exposed to impulsive sound took longer to recover (return to swimming closer to the water's surface) compared to continuous noise [26]. This suggests that the temporal structure of a sound source, as well as the frequencies and amplitude of it, could have an important influence on behavioural responses to that source. Further work is needed on how the temporal, frequencies, and amplitude of anthropogenic noise sources affect behaviour.

Pile-driving playback decreased the cohesiveness of seabass groups, which is the opposite to what would be expected if the fish treated the additional noise as a predation threat [57]. Under predation threat, groups are expected to become more cohesive as individuals reduce risk through dilution and confusion effects [74–78]. Instead, fish in our experiment increased their distance to the group's centroid and between nearest neighbours. We also found that the speed and directional coordination of nearest neighbours decreased more in the pile-driving playback compared to the ambient-sound playback. Again, this is the opposite to what would be expected under increased predation threat, where individuals should be highly sensitive and coordinate their movements more strongly with near neighbours [48,49]. Instead, our findings are consistent with the idea that pile-driving playback disrupts the cohesion and coordination of individuals in the shoals.

Coordination of the movements of individual fish is thought to be modulated primarily by lateral-line and visual sensory inputs [41,46]. If detection of nearest-neighbours' movements through the lateral line were obstructed by the playbacks, this could explain reductions in directional and speed correlations between nearest neighbours. This would effectively be an example of masking, a uni-modal effect of additional noise, although that has mostly been considered to date with respect to vocal communication [79]. Alternatively, even if lateral-line information was not disrupted, additional noise may still have impacted the ability of individuals to process sensory information through cross-modal effects. Cross-modal effects occur when the processing and effective use of information is negatively affected by additional noise as a consequence of stress and/or distraction [15]. These cross-modal effects have recently been demonstrated in other species [15] and are well known in the cognitive sciences [80]. Therefore, cross-modal effects could also occur when attempting to coordinate movement with near neighbours, ultimately affecting the structure of these groups. It may be possible to assess whether uni-modal, cross-modal, or both effects influence the schooling behaviour of fish by knocking out the functionality of the lateral-line system using aminoglycoside antibiotics [42]. By then assessing whether the schooling behaviour of fish was impacted further by the addition of noise, this would provide evidence that noise impacts schooling behaviour even if sound could not be detected with the lateral-line system. More generally, cross-modal effects can be assessed by measuring whether behavioural responses to stimuli that have no auditory component (e.g. visual cues or olfactory cues) are impacted by sound, as has recently been investigated [15].

Disruption to how individuals interact in groups could impact some of the associated benefits of group living, including a reduced predation risk and access to social information [27,28]. Individuals in less cohesive groups are attacked more frequently than individuals in more cohesive groups [78], and we observed that individuals increased their distances between one another more in response to the pile-driving compared to the ambient-sound playback. Individual fish in shoals also gain information from others, for example, about a detected threat, by copying the speed and directional changes of near neighbours [49,81]. Similarly, the collective ability of groups to sense complex gradients in their environment is modulated by how individuals copy the speed changes of others [50]. How individuals respond to each other's movements determines the likelihood and extent of information propagation in animal groups [82], and disruptions by anthropogenic noise could have considerable ecological implications for group-living species. Non-lethal effects such as these are crucial for our understanding of how noise pollution impacts the behaviour and survival of animals, and this will be important to consider for animals in natural conditions with real sound sources.

Our experiment focused on responses to a single relatively short-term noise exposure, as is the case with the majority of fish research to date (see [53,83] for exceptions). For a full understanding of the impacts of anthropogenic noise, longer-term studies are also needed, because animals may be able to compensate during quieter periods and responses may change with repeated or chronic exposure [53,84–86]. Ideally, those future studies should be conducted in natural conditions with real-world sound sources [16] to ensure maximum ecological and acoustic validity [55]. However, captive experiments such as ours do provide a valuable stepping stone in the study of environmental stressors, including noise [16,34,87], not least because of the ability to control tightly the conditions and to collect detailed individual-based data.

Our work highlights the potential for noise from anthropogenic sources to disrupt the coordination of shoaling fish. Whether this translates to functional consequences for fishes, such as changes in feeding success or predation risk, will now need to be assessed. Nevertheless, our results demonstrate that sound can influence the spatial and directional organizational characteristics of fish shoals, and as such, should be considered when environmental impact assessments of construction projects in marine or freshwater environments are conducted.

## Data accessibility

All data accompanying this paper can be downloaded from: https://figshare.com/s/237fda89f8ffc265a6f6.
